# A weakly supervised deep learning model integrating noncontrasted computed tomography images and clinical factors facilitates haemorrhagic transformation prediction after intravenous thrombolysis in acute ischaemic stroke patients

**DOI:** 10.1186/s12938-023-01193-w

**Published:** 2023-12-19

**Authors:** Xiaoshuang Ru, Shilong Zhao, Weidao Chen, Jiangfen Wu, Ruize Yu, Dawei Wang, Mengxing Dong, Qiong Wu, Daoyong Peng, Yang Song

**Affiliations:** 1 Department of Radiology, Central Hospital of Dalian University of Technology, No. 826 Xinan Rd, Shahekou District, Dalian, 116033 Liaoning Province China; 2 Department of Radiology, Affliated ZhongShan Hospital of Dalian University, No. 6 Jiefang Rd, Zhongshan District, Dalian, 116001 Liaoning Province China; 3 InferVision Medical Technology Company Ltd, 25F, Building E, Yuanyang International Center, Chaoyang District, Beijing, 100025 China; 4 Department of Neurology, Central Hospital of Dalian University of Technology, No. 826 Xinan Rd, Shahekou District, Dalian, 116033 Liaoning Province China

**Keywords:** Noncontrast computed tomography, Haemorrhagic transformation, Deep learning, Machine learning, Ischaemic stroke

## Abstract

**Background:**

Haemorrhage transformation (HT) is a serious complication of intravenous thrombolysis (IVT) in acute ischaemic stroke (AIS). Accurate and timely prediction of the risk of HT before IVT may change the treatment decision and improve clinical prognosis. We aimed to develop a deep learning method for predicting HT after IVT for AIS using noncontrast computed tomography (NCCT) images.

**Methods:**

We retrospectively collected data from 828 AIS patients undergoing recombinant tissue plasminogen activator (rt-PA) treatment within a 4.5-h time window (*n* = 665) or of undergoing urokinase treatment within a 6-h time window (*n* = 163) and divided them into the HT group (*n* = 69) and non-HT group (*n* = 759). HT was defined based on the criteria of the European Cooperative Acute Stroke Study-II trial. To address the problems of indiscernible features and imbalanced data, a weakly supervised deep learning (WSDL) model for HT prediction was constructed based on multiple instance learning and active learning using admission NCCT images and clinical information in addition to conventional deep learning models. Threefold cross-validation and transfer learning were performed to confirm the robustness of the network. Of note, the predictive value of the commonly used scales in clinics associated with NCCT images (i.e., the HAT and SEDAN score) was also analysed and compared to measure the feasibility of our proposed DL algorithms.

**Results:**

Compared to the conventional DL and ML models, the WSDL model had the highest AUC of 0.799 (95% CI 0.712–0.883). Significant differences were observed between the WSDL model and five ML models (*P* < 0.05). The prediction performance of the WSDL model outperforms the HAT and SEDAN scores at the optimal operating point (threshold = 1.5). Further subgroup analysis showed that the WSDL model performed better for symptomatic intracranial haemorrhage (AUC = 0.833, F1 score = 0.909).

**Conclusions:**

Our WSDL model based on NCCT images had relatively good performance for predicting HT in AIS and may be suitable for assisting in clinical treatment decision-making.

**Supplementary Information:**

The online version contains supplementary material available at 10.1186/s12938-023-01193-w.

## Background

Stroke is the second leading cause of mortality and a major cause of disability worldwide [1]. Acute ischaemic stroke (AIS) is the most common type, accounting for 69.6–70.8% of strokes [2]. At present, the prognosis of AIS patients can be significantly improved by reperfusion therapy, such as intravenous thrombolysis (IVT) with recombinant tissue plasminogen activator (rt-PA) and endovascular thrombectomy [3, 4]. However, intracranial haemorrhagic transformation (HT), especially symptomatic intracranial haemorrhage (SICH), after IVT remains the most dreaded complication, as it can lead to a lifelong deterioration of neurological function and even death [5]. Epidemiological investigations show that the incidence of HT after IVT in patients with AIS is 1.70–10.30% [6, 7]. The incidence of this complication can be reduced by accurate and efficient identification of individuals at risk. Therefore, accurate and timely prediction of the risk of HT before IVT may change the treatment decision and improve the clinical prognosis [8].

The roles of medical imaging in diagnosing AIS are expanding rapidly, and blood‒brain barrier permeability studies via computed tomography perfusion (CTP) imaging and magnetic resonance imaging (MRI) have a high sensitivity for predicting HT [9, 10]. However, MRI scans are not part of the routine imaging procedures in emergency green channel settings in most hospitals. Computed tomography (CT) imaging procedures, including noncontrast computed tomography (NCCT), computed tomography angiography (CTA), and CTP, are the first choice for AIS diagnosis and are important in HT prediction. However, CTA and CTP are time-consuming and limited by contraindications, and they are not readily available in most grassroots hospitals. Thus, these two imaging methods for predicting HT are still far from clinical use. NCCT, due to its relatively high speed, broad accessibility and cost-effectiveness compared with MRI and CTP, is most widely used in the emergency settings. Therefore, HT prediction based on NCCT may be the most practical application direction. However, little information for HT prediction can be detected visually on NCCT. It has been reported that neuroimaging signs based upon acute NCCT scans can predict HT after thrombolytic therapy, including visible acute cerebral ischaemic lesions, hyperdense cerebral artery signs, leukoaraiosis, and calcification in the main cerebral vessels [11, 12]. However, some features, such as hypoattenuation of the middle cerebral artery territory, are difficult to visually detect on NCCT, and its detection is highly dependent on the raters’ experience, resulting in inaccurate quantification and significant interrater variability [13, 14]. Furthermore, HT after IVT is a complex pathophysiological process that can be predicted not only by imaging changes but also by clinical data and biochemical indicators. Therefore, comprehensive consideration of imaging and clinical information is appropriate for clinical settings. However, the existing scales to assess the risk of HT after thrombolysis, including the Haemorrhage After Thrombolysis (HAT) score [15], SEDAN score [16], Multicentre Stroke Survey (MSS) score [17], Safe Implementation of Treatments in Stroke (SITS) score [18], and GRASPS score [19], have limitations and disadvantages. The predictive value of these scores is limited by the variation in sample populations, the diagnostic ability of radiologists, and the complexity of all the factors that are involved, resulting in the predictive value varying amongst different studies [19–21], which implies a certain degree of inaccuracy. Therefore, it is imperative to develop a more reliable and effective tool for the early and timely prediction of HT in AIS patients after IVT.

Deep learning (DL), a subfield of machine learning (ML), has provided state-of-the-art algorithms for medical image recognition with the advantage of automated featurization [22, 23]. DL methods have been used to diagnose and predict final stroke lesion volume, tissue outcome, and treatment effect based on MRI images [24, 25]. DL methods have also been applied to predict clinical functional outcomes following reperfusion therapy for AIS using radiological image data [26–28]. Notably, most applications of DL are currently based on supervised learning with a large number of training samples that are strictly and meticulously annotated [29]. However, the general labelling of included image data with strong supervision information is difficult to perform due to the requirements of the intensive labour force. To tackle this problem, weakly supervised learning methods emerged using coarse-grained labels and so on. It is noteworthy that multiple instance learning (MIL), which is a typical weakly supervised learning method, has shown great advances in medical imaging analysis [30, 31]. Due to the requirements of numerous data needing meticulous annotation, it is difficult for conventional DL algorithms to achieve better performance in predicting HT after IVT in AIS patients. However, weak supervision may be a potential method to solve the problem under the current situation. Recently, no weakly supervised learning-based DL algorithm for predicting HT using NCCT images has been reported [32, 33].

In this study, we developed a fully automated DL framework for predicting the HT of AIS patients based on baseline NCCT images and clinical risk factors. We aim to provide an alternative, reliable, and convenient method using available data at admission and to assist in the clinical selection of patients suitable for thrombolysis. To address the data problems of indiscernible features and imbalanced samples, weakly supervised methods of multiple instance learning (MIL) and active learning were added. To verify the efficacy of the weakly supervised deep learning (WSDL) model, we compared the WSDL model with the conventional baseline DL model, various ML models, and the existing HT risk assessment scales (HAT and SEDAN score), which were related to NCCT images.

## Results

### Baseline clinical features and data characteristics

**Fig. 1:**
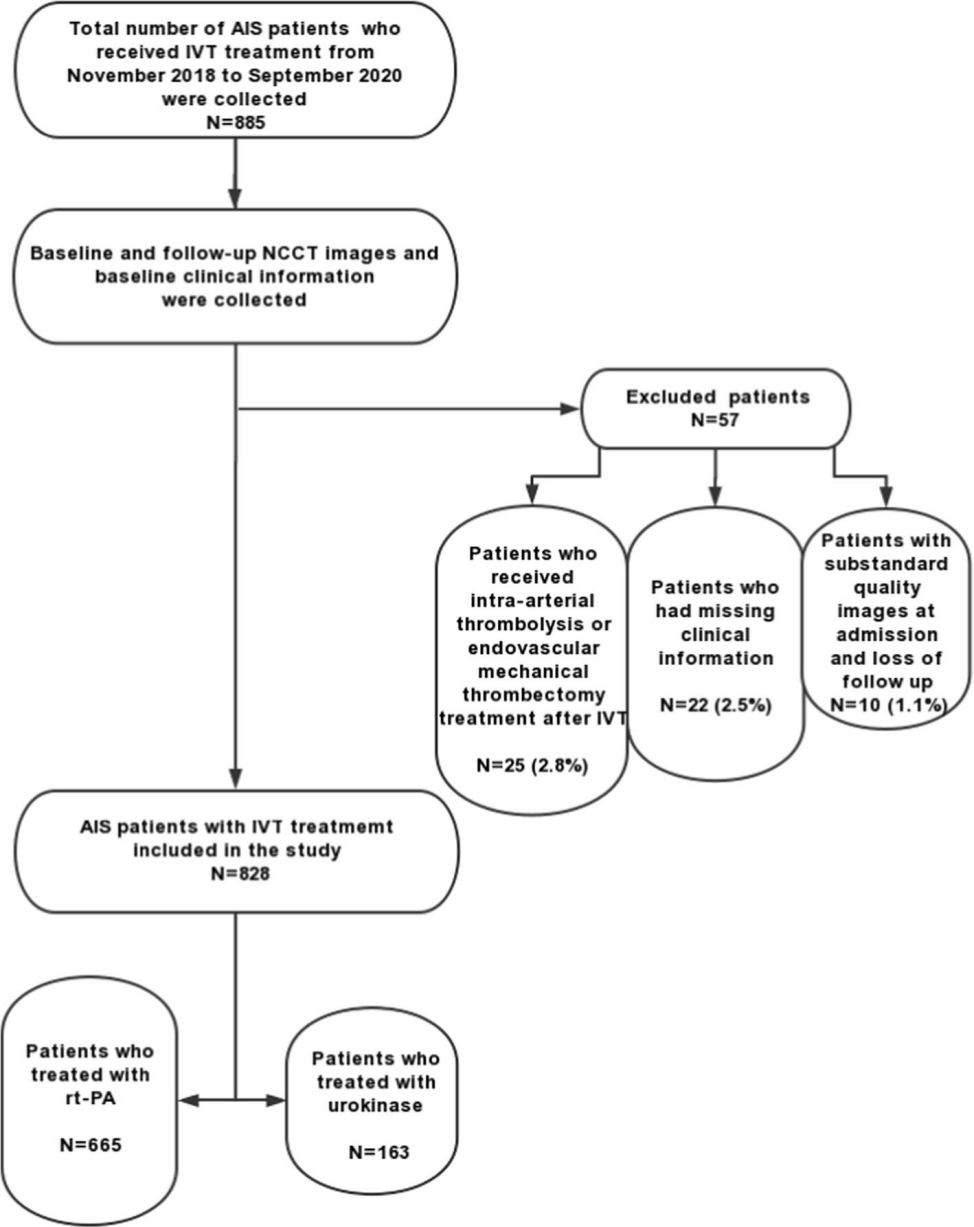
Flow diagram of the dataset selection process for eligible patients

A total of 885 patients with AIS who received IVT were enrolled in this study. The flow diagram of patient inclusion is shown in Fig. 1. After the exclusions, data for 828 patients were used in the final analysis. The patients were split into the HT group (positive sample, *n* = 69, 8.3%) and the non-HT group (negative sample, *n* = 759, 91.7%). The baseline clinical features of patients in the HT group and non-HT group are shown in Table 1. Age, atrial fibrillation, diabetes mellitus diagnosis, glucose level, and NIHSS score on admission between the two cohorts were statistically significant (*P* < 0.05).

**Table 1:** The baseline clinical features of patients in the HT group and non-HT group

Characteristics	HT (*n* = 69)	Non-HT(*n* = 759)	*P* value
Age (year) (median, IQR)	70 (62–82)	67 (59–77)	0.022
Male, *n *(%)/female, *n* (%)	46 (66.7)/23 (33.3)	501 (66.0)/258(34.0)	0.912
rt-PA, *n* (%)/urokinase, *n* (%)	57 (82.6)/12 (17.4)	608 (80.1)/151 (19.9)	0.617
Hypertension, *n* (%)	49 (71.0)	569 (75.0)	0.470
Diabetes mellitus, *n* (%)	36 (52.2)	302 (39.8)	0.045
Atrial fibrillation, *n* (%)	29 (42.0)	146 (19.2)	0.000
Hypercholesterolemia, *n* (%)	18 (26.1)	139 (18.3)	0.115
Currently smoking, *n* (%)	26 (37.7)	374 (49.3)	0.065
Previous stroke, *n* (%)	46 (66.7)	487 (64.2)	0.678
Antiplatelets/Anticoagulation, *n* (%)	21 (30.4)	167 (22.0)	0.109
Glucose level (mmol/L) (median, IQR)	7.5 (5.9–9.5)	6.4 (5.3–8.4)	0.015
Systolic BP (mmHg) (median, IQR)	150 (130–169)	157 (140–170)	0.138
NIHSS score on admission(median, IQR)	12 (6–18)	4 (2–8)	0.000
OTT (min) (median, IQR)	120 (60–188)	120 (60–180)	0.640
PLT count (10^9^/L) (median, IQR)	199 (158–247)	206 (170–245)	0.397
Body temperature (°C) (median, IQR)	36.5 (36.5–36.6)	36.5 (36.4–36.5)	0.406

*HT* haemorrhagic transformation, *IQR* interquartile range, *BP* blood pressure, *NIHSS* National Institutes of Health Stroke Scale, *OTT* symptom onset to treatment time, *PLT count* platelet count

Continuous variables were expressed as medians with corresponding interquartile ranges and categorical variables were described as proportions. Continuous variables were compared using the Mann–Whitney *U* test for non-normally distributed and differences in categorical variables were assessed by the chi-squared test or Fisher’s exact test between the HT and non-HT patient groups.

### Performance comparison of the WSDL model with ML models

**Fig. 2:**
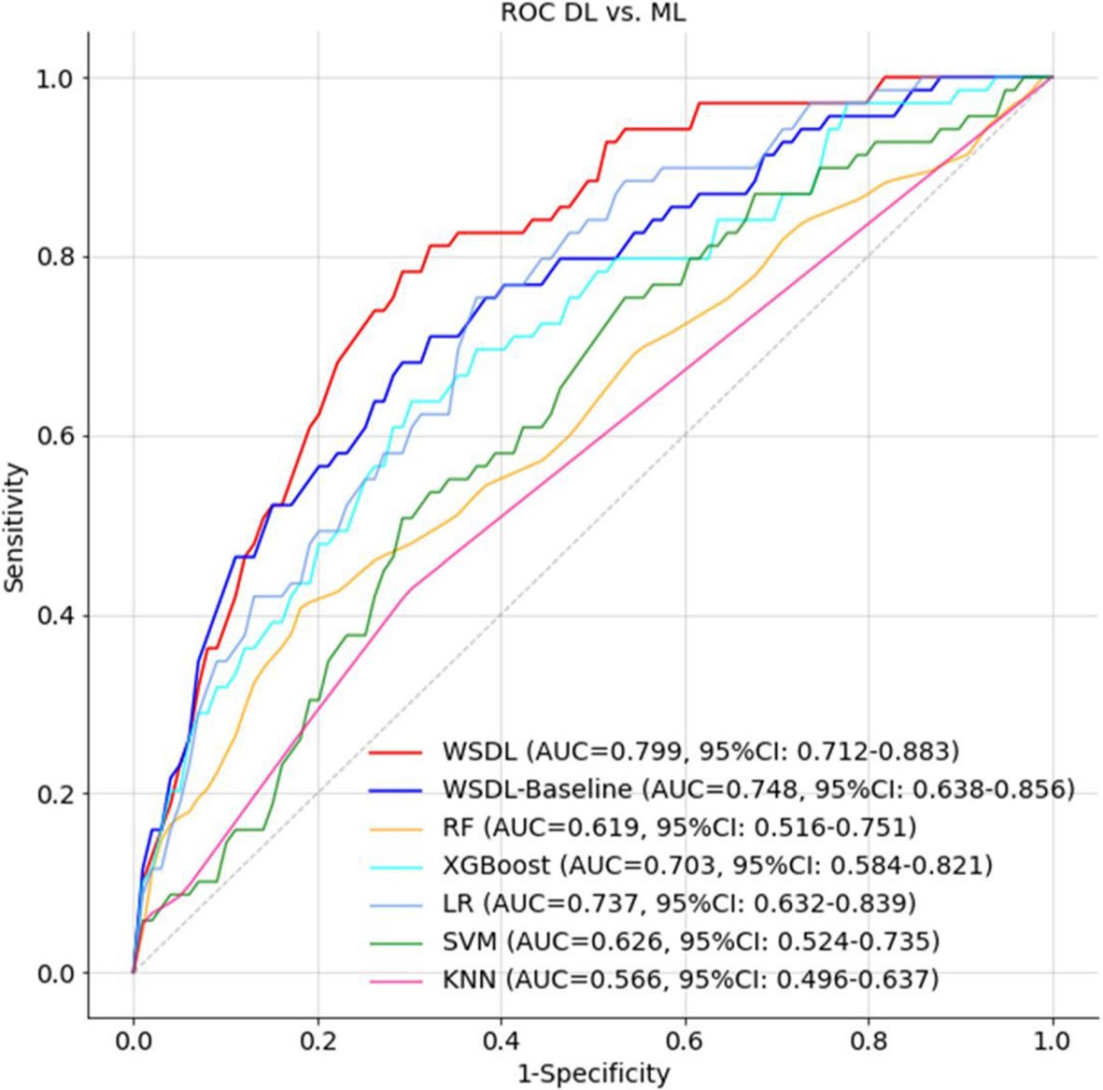
Illustration of the accuracy in terms of ROC curves for various predictive models of HT based on NCCT imaging data and clinical information

As mentioned, the WSDL model was proposed and developed in this study to address the problems of indiscernible features, imbalanced data, and needed intensive labelling force. As a comparison, a baseline DL model and five ML models were also developed to predict HT after IVT. The following five ML models were used: support vector machine (SVM), logistic regression (LR), k-nearest neighbours (KNN), random forest (RF), and eXtreme gradient boosting (XGBoost). As shown in Fig. 2 and Table 2, after being trained with the coarse-grained labels, the WSDL and baseline-DL models showed better performance than the ML models. In particular, the WSDL model achieved the highest AUC value of 0.799 (95% CI 0.712–0.883). There were significant differences in the AUCs between the WSDL model and the SVM, KNN, RF, and XGBoost models (*P* < 0.05, DeLong test), whereas there was no significant difference between the WSDL model and the LR model (*P* > 0.05, DeLong test). At the operating point of a fixed sensitivity of 0.8 and fixed specificity of 0.7, the other indicators for the WSDL model were superior to those of the other models.

**Table 2:** Performances of all the models

	AUC	High sensitivity operating point			High specificity operating point		
		Sensitivity	Specificity	Accuracy	Sensitivity	Specificity	Accuracy
WSDL	0.799 (0.712,0.883)	0.826 (0.669,0.980)	0.635 (0.576,0.694)	0.651 (0.596,0.706)	0.783 (0.609,0.942)	0.701 (0.642,0.756)	0.708 (0.653,0.760)
baseline-DL	0.748 (0.638,0.856)	0.826 (0.671,0.984)	0.518 (0.457,0.579)	0.544 (0.486,0.601)	0.681 (0.496,0.869)	0.701 (0.642,0.758)	0.699 (0.644,0.754)
LR	0.737 (0.632,0.839)	0.826 (0.670,0.980)	0.557 (0.497,0.618)	0.579 (0.523,0.636)	0.609 (0.407,0.807)	0.701 (0.646,0.758)	0.693 (0.639,0.749)
XGBoost	0.703 (0.584,0.821)	0.826 (0.678,0.981)	0.380 (0.324,0.437)	0.418 (0.364,0.471)	0.638 (0.456,0.835)	0.701 (0.644,0.757)	0.695 (0.641,0.750)
SVM	0.626 (0.524,0.735)	0.826 (0.670,0.980)	0.302 (0.251,0.351)	0.346 (0.297,0.393)	0.391 (0.238,0.566)	0.701 (0.643,0.755)	0.675 (0.620,0.727)
RF	0.619 (0.516,0.751)	0.826 (0.671,0.982)	0.315 (0.259,0.370)	0.358 (0.305,0.410)	0.464 (0.27,0.659)	0.729 (0.675,0.782)	0.707 (0.655,0.758)
KNN	0.566 (0.496,0.637)	0.638 (0.512,0.761)	0.506 (0.472,0.541)	0.517 (0.485,0.550)	0.638 (0.512,0.761)	0.506 (0.472,0.541)	0.517 (0.485,0.550)

### Performance comparison of WSDL model with HAT and SEDAN score

**Fig. 3:**
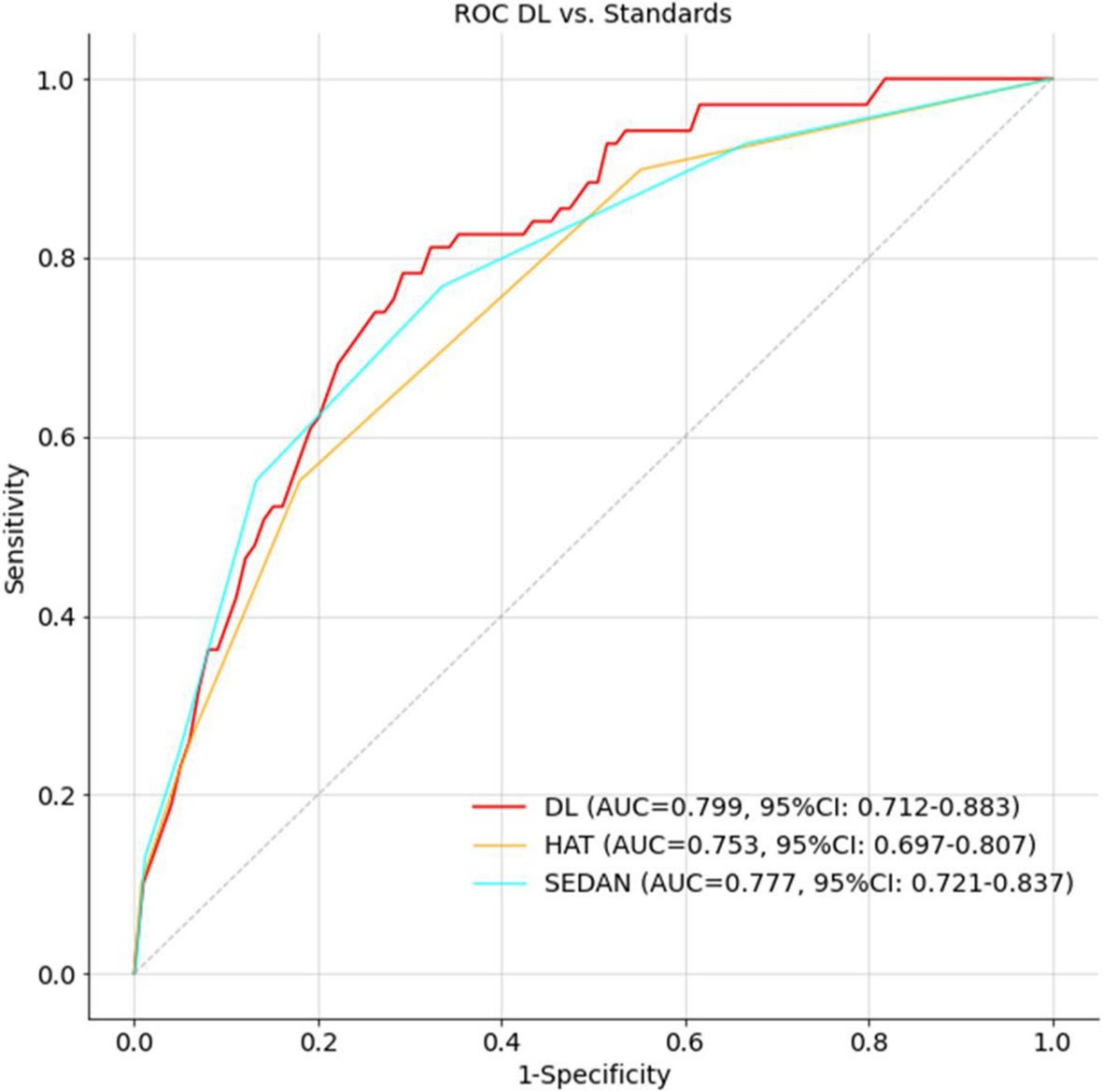
Illustration of the accuracy in terms of ROC curves for WSDL model and HAT and SEDAN scores of HT based on NCCT imaging data and clinical information

The performance of the WSDL model and current established clinical prognostic tools (HAT and SEDAN score) were evaluated and are shown in Fig. 3 and Table 3. The prediction performance of the WSDL model outperforms the HAT and SEDAN scores at the optimal operating point (threshold = 1.5), except the HAT score shows higher accuracy and specificity. Notably, the AUC (0.799, 95% CI 0.712–0.883) and sensitivity (79.7%, 95% CI 63.2–95.5%) of our WSDL model were higher than both the AUC value for the HAT and the SEDAN (0.753 and 0.777, respectively) as well as the sensitivity values for both scores (55.1% and 76.8%, respectively).

**Table 3:** Performances of WSDL model and HAT and SEDAN score

	AUC	Accuracy	Sensitivity	Specificity	PPV	NPV
WSDL	0.799 (0.712,0.883)	0.735 (0.683,0.787)	0.797 (0.632,0.955)	0.730 (0.674,0.784)	0.213 (0.163,0.263)	0.976 (0.956,0.994)
HAT	0.753 (0.697,0.807)	0.797 (0.771,0.824)	0.551 (0.438,0.66)	0.819 (0.793,0.847)	0.217 (0.174,0.260)	0.953 (0.941,0.964)
SEDAN	0.777 (0.721,0.837)	0.673 (0.642,0.704)	0.768 (0.670,0.872)	0.664 (0.632,0.697)	0.172 (0.150,0.196)	0.969 (0.957,0.983)

### Visualization of the regions on which the WSDL model focussed using gradient-weighted class activation mapping (Grad-CAM)

**Fig. 4:**
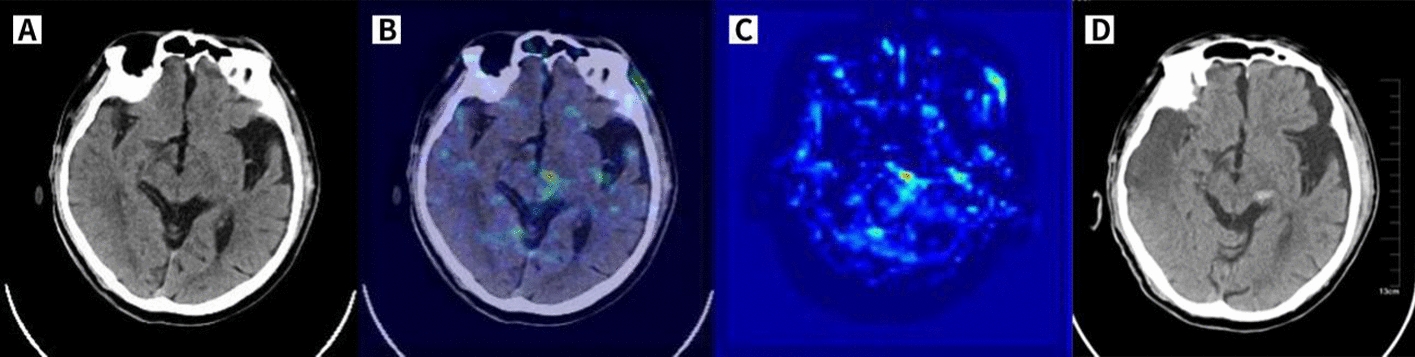
**CAM of a correctly predicted HT case**, as shown in **C**. The patient was admitted to our hospital with sudden inactivity of the left limb for 2 h, and haemorrhage and clear infarct lesions were not found on baseline NCCT (**A**). Then, the patient was given rt-PA 56 mg. The cranial NCCT was re-examined within 24 h, and there were HTs in the left thalamus and midbrain cerebral peduncle (**D**). Superimposing the heatmap on the native image (**B**) highlights the left thalamus and midbrain cerebral peduncle, which were the regions of HT that occurred after IVT (as shown by the arrow), thus proving that the model predicting upcoming HT was favourable

The activation of the WSDL model when predicting HT was mainly focussed on the brain tissue region related to infarct lesions, small-vessel ischaemia, leukoaraiosis, and atrophy or the location where HT would occur in most cases. Figure 4 shows a typical HT case to visualize the class activation maps (CAMs).

### Performance of the WSDL model in HT subgroups

The patients were categorized into three groups (patients without ICH, *n* = 759; patients with SICH, *n* = 6; patients with asymptomatic ICH, *n* = 63) based on clinical outcome, and subgroup analyses were conducted (Table 4). The WSDL model performed better for SICH, reaching an AUC of 0.833 (95% CI 50.0–100.0%) and an F1 score of 0.909 (95% CI 66.7–100.0%).

**Table 4:** Performance of the WSDL model in HT subgroups

	Accuracy	Accuracy 95% CI	F1	F1 95% CI
Without ICH	0.686	(0.655,0.721)	0.814	(0.791,0.838)
Asymptomatic ICH	0.619	(0.508,0.73)	0.765	(0.674,0.844)
SICH	0.833	(0.5,1)	0.909	(0.667,1)

## Discussion

We used a WSDL model to predict HT risk in AIS patients with baseline NCCT and clinical risk factors. The WSDL model demonstrated good discriminatory ability compared with the baseline DL model, five ML models, and existing clinical prognostic tools (HAT and SEDAN scores), and especially exhibited a high performance in predicting SICH. This study showed that HT risk prediction could be achieved conveniently with the WSDL method based on the limited admission data before IVT.

Classical ML methods have been developed for HT prediction using clinical information [34–38]. However, DL algorithms and NCCT have not been used to predict the risk of HT in AIS patients. Most previous ML models were based on the Electronic Health Record dataset or used only structured data [35–38]. However, the prediction performance was unsatisfactory or less practical in the clinic. For example, Wang et al. used a public dataset to build an LASSO logistic regression prognostic model predicting symptomatic HT that achieved a mean external AUC of 0.71 [35]. The study selected 612 risk predictors as inputs for the model, which are difficult to collect for routine clinical diagnosis and treatment; thus, the method is less practical. The clinical information used in our WSDL model is readily available within a few minutes of a patient’s arrival at the emergency room in almost all medical centres. It can be embedded into CT image analysis software for HT prediction, and the HT warning will be automatically given immediately after the CT examination is completed, which is more practical and has crucial application value in clinical settings. Consequently, our method could provide an efficient and easy-to-use solution for assisting clinical decision-making.

The clinical factors included in our study are readily available and critical for predicting HT after IVT. Many risk factors have been previously confirmed to be associated with HT after IVT [11, 39, 40], including age, the severity of the stroke, baseline glucose, the presence of atrial fibrillation, diabetes, hypertension, previous cerebral vascular diseases or ischaemic heart disease, congestive heart failure, renal dysfunction, use of antiplatelet drugs or statins, leukoaraiosis, and early signs of infarction on head CT. Consistently, similar risk factors were also observed in our study and selected for model development. Of note, in addition to those factors (age, atrial fibrillation, NIHSS score, and glucose level on admission) that were previously confirmed as the most important independent predictors for individualized HT prediction [39, 40], we included more clinical information, which might provide more predictive information than the HAT and SENDA scores. Moreover, existing HT prognostic models cannot address the full complexity of all the factors that are involved, the current feature selection method is to explore the linear relationship between factors, and it is difficult to capture the nonlinear relationship. In this study, our DL model learned these clinical factors using a convolutional neural network with a more powerful feature extraction ability and fully explored their interactive relationship.

Previous studies indicated that the HAT and SENDA played key roles in predicting HT after IVT [6, 20, 21], and it is worth noting that the two scores also involve CT signs. Therefore, the DL model we developed was compared with the conventional two scores to verify its efficacy in HT prediction. In this study, our proposed DL model utilizing raw CT images and essential clinical information outperformed these standard prognostic scores. We noticed that the HAT and SENDA scores involve early infarct signs on CT and hyperdense cerebral artery signs for the individualized prediction of HT; however, these scores are limited by the use of dichotomization/categorization of predictors and may decrease the predictive accuracy. In sharp contrast, our DL model used whole-brain images as its input rather than selected subparts, given that certain brain CT background appearances related to small-vessel ischaemia, leukoaraiosis, previous strokes, and atrophy were recognized predictors of HT [11, 41, 42]. Our proposed DL model was able to predict HT occurring remotely from the infarcted territory, which also implied that background brain features might be just as important as focal CT markers of acute ischaemia, which was consistent with a previous research report [14].

Some studies have constructed HT prediction models using medical images and multiple ML methods [14, 43–45]. Yu et al. developed multiple ML models and long short-term memory (LSTM) models for predicting HT based on MRI perfusion- and diffusion-weighted images [43, 44]. The regression model performed best with an accuracy of 83.7 ± 2.6%, and the LSTM model reached an AUC-ROC of 89.44%. Although multiparametric MRI offers more information about stroke pathophysiology, MRI takes longer than CT scans, which may delay the treatment of critically ill patients. NCCT, due to its speed and limited contraindications, is most widely used in the emergency green channel diagnosis and treatment of AIS. Bentley et al. [14] collected CT images and clinical features of patients with AIS and IVT and constructed an SVM model to predict SICH. However, the prediction performance was unsatisfactory, with an AUC of 0.744, which was lower than our DL model. In addition, the sample size was relatively small (training sets: *n* = 106, test sets: *n* = 10), which to some extent weakened the robustness of their results. In addition, the developed ML model also worked via a complex process, including drawing the region of interest, feature definition, feature reduction, and sample inference, which hinders its practical use in routine clinical diagnosis.

The innovative algorithm design is critical to the success of our WSDL model. In this study, we used the weakly supervised MIL method and active learning algorithm to cope with inherent data problems. MIL helps to address the difficulty in labelling ambiguous edges and labour costs. Because AIS lesions tend to be missed in NCCT images, lesion identification was achieved using MIL by the cross-combination of the image slices integrating varied window widths and centres instead of elaborate lesion outlines. Compared to conventional DL and radiomics methods, MIL could reduce the bias induced by radiologists’ experience and improve model generalizability. Because HT is related to collateral circulation and white matter lesions, inputting the whole-brain image also helps to extract more HT information for the DL model. Although the MIL method has relevant applications in COPD or glioma [46, 47], this is the first study to apply MIL to HT identification. Active learning-based active smoothing loss (AS loss) improved the model’s ability to identify outcome-related features and increase the importance weight of reliable positive cases by increasing the weight of the effective features. We used the idea of active learning and selected high-quality annotation samples online for feature learning during model training, which greatly improved the generalization ability of the model and addressed the data imbalance problem.

Our retrospective study has some limitations that need to be addressed. The number of positive sample cases in this study is small. This increases the risks of model overfitting and thus affects the model performance, although the proposed model was designed to solve the problem of a small, skewed dataset. The number of cases with SICH was also small due to the limited incidence of the condition. Therefore, studies involving larger samples of HT, especially SICH, are needed to validate and optimize the DL model, and the results for SICH should be interpreted with caution. In addition, the standard of HT used in the study is NCCT 24 h after IVT, which may underestimate the ratio of HT because the haemorrhage time and volume would influence the result; therefore, susceptibility weighted imaging (SWI) would be included as the standard in future work. In addition, this is a retrospective study from a single institution. A multicentre prospective study is warranted to validate the generalization ability of the model. Moreover, only NCCT images and clinical risk factors were used in this study. Incorporating other imaging modalities may improve the model’s performance further; previous non-DL studies have shown that HT prediction could be improved by including CTP and reperfusion data [9]. We are planning to investigate this in future work.

## Conclusions

We constructed a DL model for predicting the risk of HT for patients with AIS after IVT based on baseline NCCT images and easy-to-collect clinical data, which is convenient for use in clinical diagnosis and treatment, especially in resource-limited areas. This information may provide a theoretical basis for clinicians to develop hierarchical follow-up and treatment plans, assist in clinical treatment decision-making, and improve the prognosis of patients with AIS.

## Materials and methods

### Study population and design

We retrospectively enrolled consecutive patients who suffered from AIS and received IVT in the emergency green channel from November 2018 to September 2020. All patients received rt-PA treatment within a 4.5-h time window or urokinase treatment within a 6-h time window. The inclusion criteria were as follows: all patients underwent baseline NCCT scans at admission, and routine follow-up NCCT scans were performed within 24 h after IVT; another NCCT scan exceeding 24 h was performed immediately in cases of rapid neurological deterioration to evaluate the presence of SICH; and the clinical data most relevant to HT were recorded. The exclusion criteria were as follows: patients who did not have baseline clinical information or for whom the imaging quality was substandard; patients who had bridging arterial thrombolysis or received endovascular mechanical thrombectomy after IVT; and patients who did not complete thrombolysis. A total of 828 patients were eligible for analysis, and 57 patients (6.4%) were excluded (Fig. 1). The protocol for this retrospective study was approved by the Ethics Committee of Dalian Municipal Central Hospital Affiliated with Dalian University of Technology, and the requirement for written informed consent was waived.

### Baseline data collection

At the time of admission, essential clinical information and baseline NCCT images were collected for each patient. Baseline information included patient demographic information (age, sex), thrombolytic drugs and dosage, past and personal medical history (hypertension, diabetes diagnosis, atrial fibrillation, current smoking status, hypercholesterolemia, previous stroke, antiplatelet, or anticoagulation therapy before enrolment), admission clinical and laboratory results (blood glucose level, blood pressure, platelet (PLT) count, temperature), baseline National Institutes of Health Stroke Scale (NIHSS) score at presentation, and time from stroke onset to treatment (OTT) (Table 1).

### Identification of intracranial HT

HT was defined as any type of ICH according to the European Cooperative Acute Stroke Study II (ECASS II) criteria [48] that could be seen on NCCT; this usually occurs within 12–36 h after IVT [49]. SICH was defined as any type of ICH on posttreatment imaging after the initiation of thrombolysis and an increase in the NIHSS score by 4 points from baseline or death (ECASS II) [7].

**Fig. 5:**
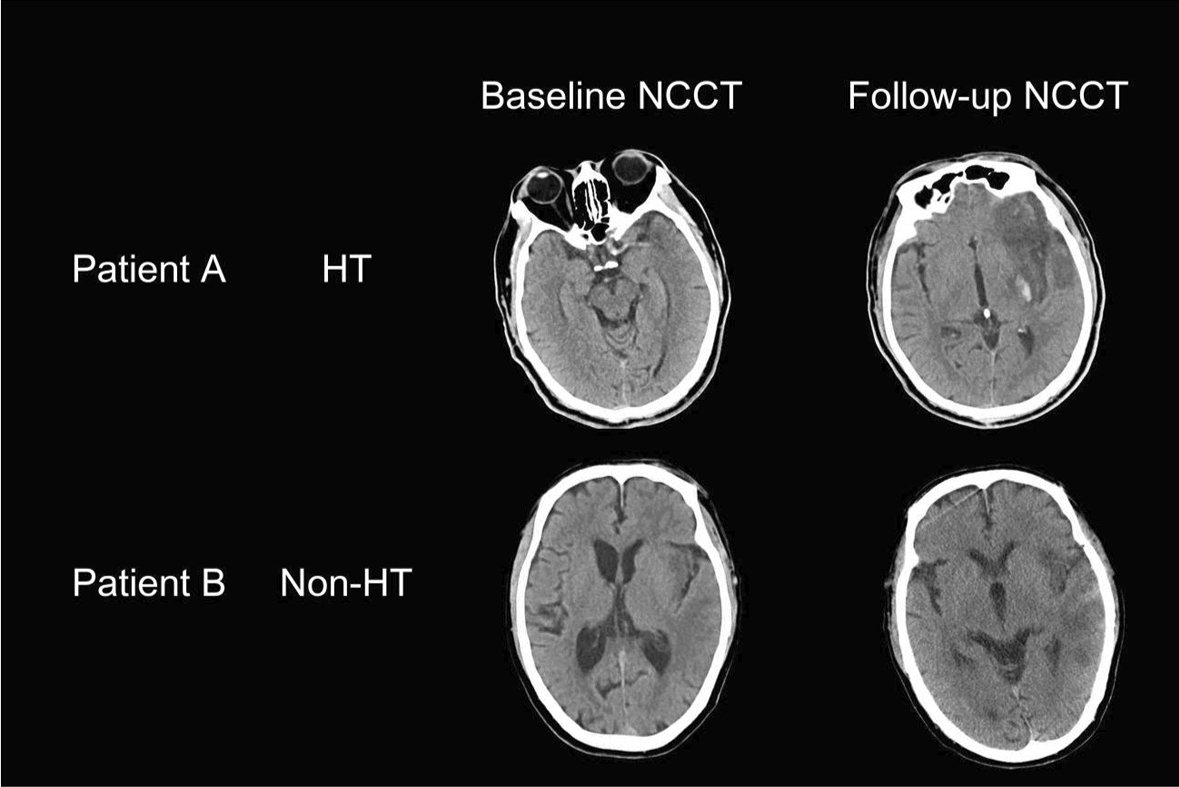
Representative pre-IVT baseline NCCT images and post-IVT follow-up NCCT images for HT and non-HT groups

The presence of HT was evaluated separately by two attending radiologists with more than 5 years of experience in neuroimaging diagnosis. When the two radiologists disagreed in evaluating the HT, they discussed until a consensus was reached. Figure 5 included representative NCCT images of HT vs non-HT groups.

### HT scores

The HAT and SEDAN scores were used for the HT score assessment. All of the patients were evaluated based on the scales by the on-duty neurologist and were recorded and proofread by a senior radiologist. Higher scores indicate a greater risk that the AIS patients would develop HT after IVT.

### Study overview and module introduction

**Fig. 6:**
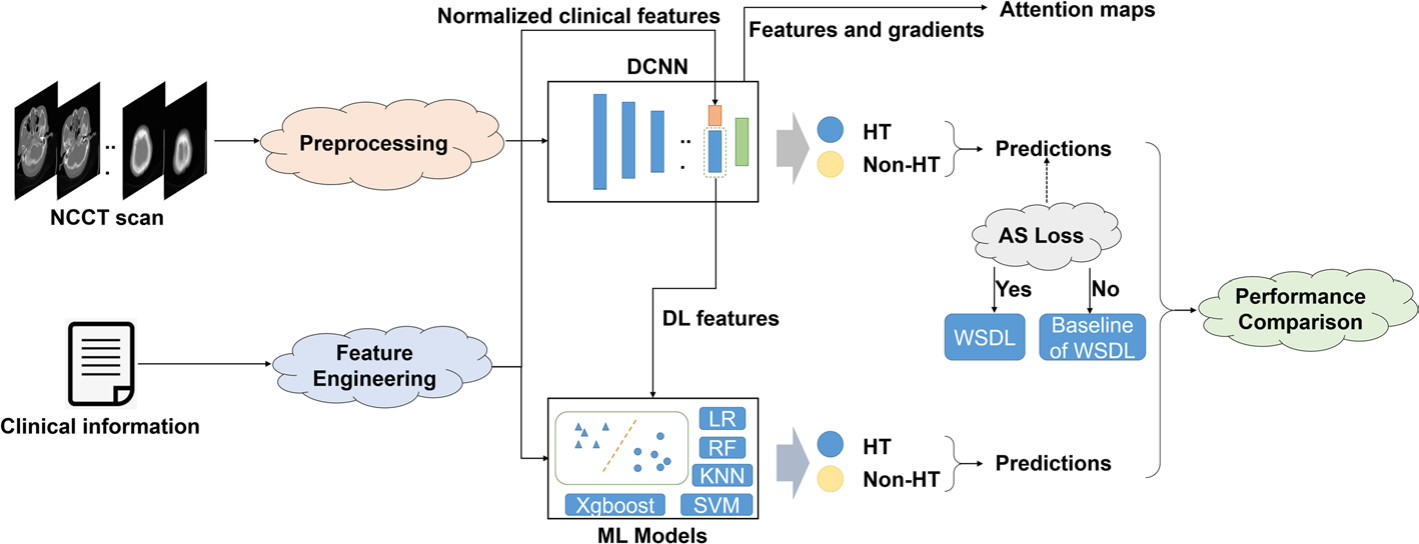
**Study overview.** This study incorporates both NCCT and clinical information for HT prediction. The WSDL model includes a pipeline of preprocessing, ImageNet pretrained dynamic convolution neural network (DCNN) and AS loss. The baseline DL was built without AS loss to output the prediction probability. For the ML models, both DL-based features and clinical information were combined with feature engineering to give the predictions. The system produces seven outputs, including predictions of five ML models, the WSDL model and the baseline of the WSDL model

An overview of the design of this study is shown in Fig. 6. The NCCT images and the clinical information were united as model inputs. A series of models were used, and the results were compared. The components are described in detail in the following section, including image preprocessing, data augmentation, our proposed WSDL model, the conventional baseline DL model, and the ML models.

### Image preprocessing

**Fig. 7:**
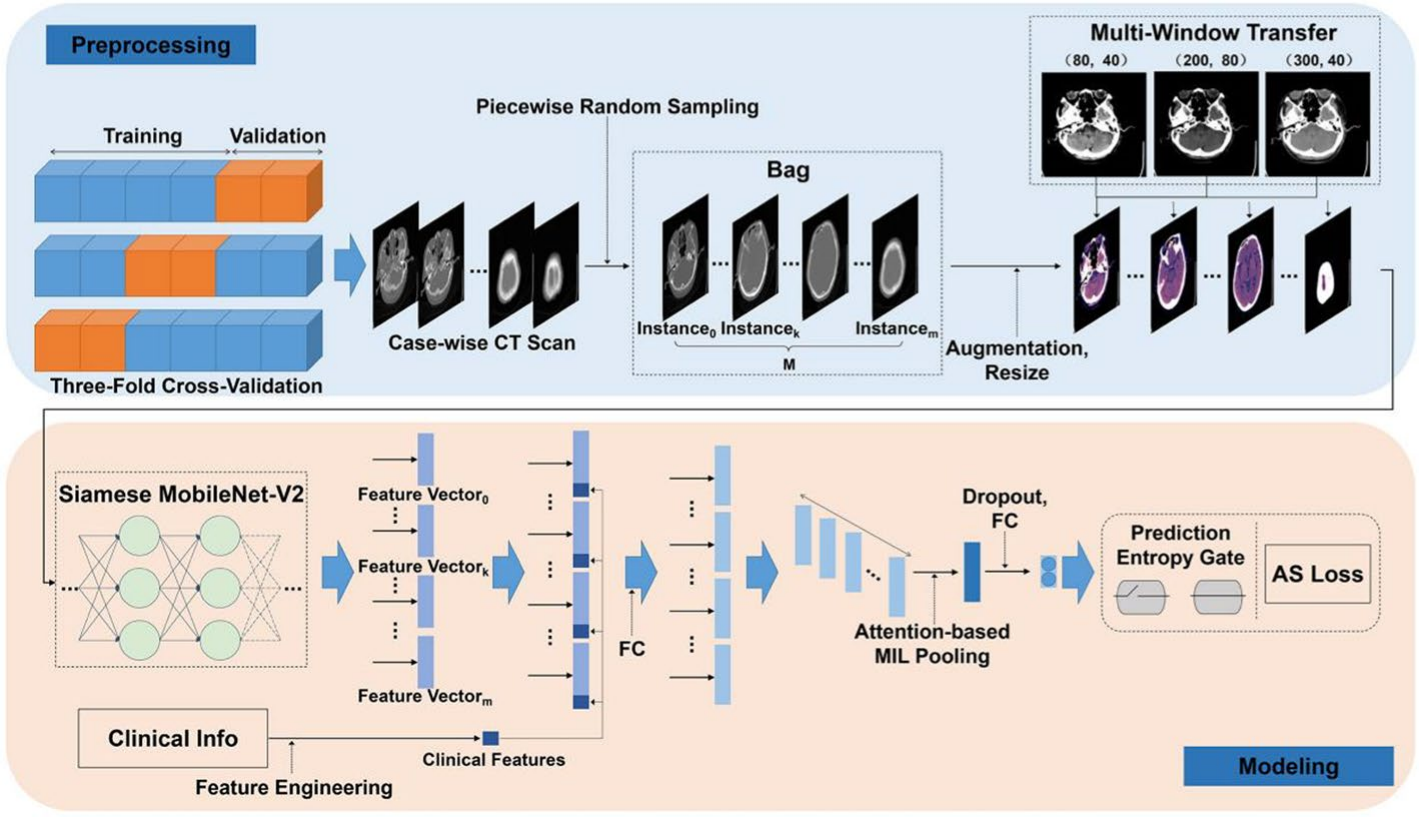
**Illustration of our WSDL framework. **Multi-instance learning and attention mechanisms were adopted to construct the model. To increase the representation information of the input image, we use the multiwindow transfer module to integrate the image information with three window widths and window levels in the channel dimension. In addition, we proposed a novel loss, i.e., AS loss, which was used during model training to ensure the classification performance

HT signs on NCCT are not obvious, and only partial slices showed positive specific information. Thus, we borrowed an idea from the MIL framework [50], a typical weakly supervised learning paradigm, to address patient-level (bag-level) prediction with no region-level annotation, as shown in Fig. 7. In the MIL setting, the CT scan was divided into M subparts with an equal height, and one slice was randomly selected from each subpart as one instance (piecewise random sampling; Fig. 7). Radiologists complete the CT image diagnosis with varying window levels and widths. To mimic this, after resampling each slice to a fixed size of 256 × 256 with INTER_NEAREST on OpenCV, we used three window widths and window levels ((W:80, L:40), (W:200, L;80), (W:300, L:40), respectively) to process the CT images, and then we stacked their outputs along the channel dimension to obtain the channel-augmented CT slices, which were the inputs of our DL model.

### Data augmentation

The imbalance of class, that is, where the number of non-HT cases is much larger than that of HT cases, is harmful to model stability. To tackle this problem, instance-level data augmentation was performed by randomly flipping in both horizontal directions and randomly scaling by uniform [0.8, 1.2]. The slices were rotated randomly by uniform [−90°, 90°] around the upright axis and uniform [−8°, 8°] around the other axis. The slices were also cropped randomly on each side by a random fraction sampled uniformly from the continuous interval [0, 0.1].

Fold 0 (total of 278 cases, 23 positive cases, and 255 negative cases), fold 1 (total of 276 cases, 23 positive cases, and 253 negative cases), and fold 2 (total of 274 cases, 23 positive cases, 251 negative cases) were used for threefold cross-validation. Threefold cross-validation and transfer learning were performed to confirm the robustness of the network.

### Our proposed WSDL model

As shown in Fig. 7, to reduce the difficulty of training and improve the convergence speed, the conventional lightweight Siamese MobileNetV2 network was used as the backbone of the DL model as the feature to obtain instance-level feature vectors of CT images. Clinical information with normalization (“gender”, “age”, “rt-PA”, “Urokinase”, “Diabetes”, “Blood Glucose”, “Smoking”, “Stroke”, “Antiplatelets/Anticoagulation”, “HBP”, “HC”, “Afib”, “PLT”, “NIHSS score”, “SBP”, “OTT”, “Temperature”) was stacked as instance-level feature vectors. The combination of the instance-level feature vectors of the NCCT images and the clinical information was passed through the fully connected layer to obtain the corresponding fusion feature vectors.

Attention-based MIL pooling was used to fuse the instance-level feature vectors, obtaining the patient-level (bag-level) feature vectors. Finally, the predicted values of HT and non-HT were output through the fully connected layer.

Attention-based MIL pooling and a loss function called AS loss based on the concept of active learning were designed, which are shown in Additional file 1.

The details of the model implementation are described in Additional file 1.

### Conventional baseline DL model and ML models

To validate the performance of our proposed WSDL model, a conventional baseline DL model without the AS loss module and five machine learning models were built.

The conventional baseline DL model was built using only the lightweight Siamese MobileNetV2 network as the backbone. The image processing and model inputs were exactly the same as our proposed WSDL model.

Five ML models were built using the combination of NCCT vectors obtained using the MobileNetV2 network and the normalized clinical features. As shown in Fig. 6, the following five ML models were used: support vector machine (SVM), logistic regression (LR), k-nearest neighbours (KNN), random forest (RF), and eXtreme gradient boosting (XGBoost). During model fitting, hyperparameters for each ML model were randomly assigned via grid search. To explore the effects of clinical information and CT images on HT identification, combinations of clinical information and DL-based features were investigated by modelling.

### Visual validation of DL diagnosis

Grad-CAM was used to identify the most important areas in distinguishing HT from non-HT [51]. Grad-CAM uses gradient information about the target class flowing into the last convolutional layer to assign importance values to each neuron and produces a localization map highlighting the important spine regions in the CT images.

### Statistical analysis

SPSS 22.0 was used for statistical analysis. Receiver operating characteristic curve (ROC) analysis was performed to obtain the area under the curve (AUC). For clinical applications, to ensure the effectiveness and practicality of the model, two operating points were chosen on the ROC curve with a sensitivity of 0.8 and specificity of 0.7. The 95% confidence interval (CI) associated with each result was obtained using the bootstrapping method [52]. The hyperparameters were optimized using nested cross-validation [43]. DeLong’s test was used to compare the AUC-ROC of each of the models, which were analysed using “R” statistical computing software (R version 3.6.3 [2020]; R Foundation for Statistical Computing). Two-tailed significance values were applied, and statistical significance was defined as *P* < 0.05.

### Supplementary Information

**Additional file 1: Table S5.** Performances of WSDL model and ML models without clinical feature modelling. **Figure S8.** Illustration of the accuracy in terms of ROC curves for WSDL model and ML models that used only NCCT feature without clinical feature modelling.

## Data Availability

The datasets used and/or analysed during the current study are available from the corresponding author on reasonable request.

## Abbreviations

WSDL: Weakly supervised deep learning
DL: Deep learning
ML: Machine learning
MIL: Multiple instance learning
HT: Haemorrhagic transformation
SICH: Symptomatic intracranial haemorrhage
IVT: Intravenous thrombolysis
rt-PA: Recombinant tissue plasminogen activator
AIS: Acute ischaemic stroke
NCCT: Noncontrasted computed tomography
CTP: Computed tomography perfusion
CTA: Computed tomography angiography
MRI: Magnetic resonance imaging
NIHSS: National Institutes of Health Stroke Scale
ECASS II: European Cooperative Acute Stroke Study II
SVM: Support vector machine
LR: Logistic regression
KNN: K-nearest neighbours
RF: Random forest
XGBoost: EXtreme gradient boosting
Grad-CAM: Gradient-weighted class activation mapping
