# Supplementary material for: A weakly supervised deep learning model integrating noncontrasted computed tomography images and clinical factors facilitates haemorrhagic transformation prediction after intravenous thrombolysis in acute ischaemic stroke patients
Source: Biomed Eng Online. 2023 Dec 19;22:129. doi: 10.1186/s12938-023-01193-w (PMC10731772; doi:10.1186/s12938-023-01193-w)
Supplement: Supplementary file 1 — Additional file 1: Table S5. Performances of WSDL model and ML models without clinical feature modelling. Figure S8. Illustration of the accuracy in terms of ROC curves for WSDL model and ML models that used only NCCT feature without clinical feature modelling. [file 12938_2023_1193_MOESM1_ESM.docx]

**Additional file**

**Study population and design**

The selection of indications and contraindications for IVT complied strictly with the 2018 guidelines for the diagnosis and treatment of acute ischaemic stroke [1]. Dosages of intravenous thrombolysis method were performed strictly according to the standard protocols [1].

**Image acquisition**

All NCCT images were acquired by a CT scanner (64-slice CT, Siemens Somatom Definition, Germany; 128-slice CT, Siemens Somatom Definition, Germany) using a helical acquisition mode and the following parameters: tube voltage of 100–120 kV; automatic tube current of 100–350 mA; matrix of 521 × 512; and slice thickness of 5 mm.

**MIL Introduction**

Specifically, we consider a group of N instances (called “Bag”) and assume that each group from a positive class sample contains at least a few slices having positive class-specific information, whereas each group from a negative class sample does not contain any slices having positive class-specific information.

We use an attention mechanism to implement the attention-based MIL pooling module with a weighted average of instances where weights are determined by a neural network.

C

**Attention-based MIL pooling**

Attention-based MIL pooling was designed to find the resonance between the instance-level feature vectors in a bag, namely, to find the most relevant embedding features for HT recognition. Attention-based MIL pooling computes the function

$M=\tanh(H)$ (1)

$\alpha=\mathrm{softmax}(w^{T}M)$ (2)

$r=H\alpha^{T}$ (3)

where *H* is a matrix consisting of output vectors [h_1_, h_2_,..., h_T_], *T* is the input sequence length, *r* is a weighted sum of the output vectors, *w* is a trained parameter vector, and *w*^T^ is a transpose.

**Active learning for the loss function**

To reduce the effect of noise labels and address the sampling imbalance dilemma, we designed a loss function called the active smoothing loss (AS loss) based on the concept of active learning. Similar to the cross-entropy loss function, AS loss uses a cross-entropy gate to control whether the sample undergoes supervised learning. Furthermore, to improve its generalization ability, the label-smoothing algorithm is integrated into the loss function. The formulas for AS loss are

 (4)

[$\hat{y}_{i}=\varepsilon(1-y_{i})+(1-\varepsilon)y_{i}$](https://fanyi.baidu.com/#zh/en/javascript:void(0);) (5)

$\delta(x)=\left\{\begin{matrix} 1,if(x\geq\mu) \\ 0,if(x<\mu) \end{matrix}\right.$ (6)

where N is the number of training samples; yi is the label of sample i, the positive class is set to 1, and the negative class is set to 0; pi is the probability that sample i is predicted to belong to the positive class; wpos and wneg are the weights of the positive and negative samples, respectively, the value range is [0, 1], and the values are set to 0.75 and 0.25, respectively; ɛ is a small modulation parameter, and the value is set to 0.1; and μ is the gate control threshold of the predicted probability, and the value is set to 0.55.

**Implementation details**

Threefold cross-validation was used to fit and validate the DL and ML models. To train the proposed WSDL model, we used Adam optimization with a batch size of 32 and a learning rate of 0.001. In addition, the Siamese MobileNet-V2 network initialized using the pretrained parameters of ImageNet [2] and dropout technique [3] were used during training. To test the DL model, we selected a middle slice from each subpart as an instance in the MIL setting to construct the input sample. During training and testing, our DL model was implemented using the popular open-source framework PyTorch and was run on an Nvidia GTX 1080Ti GPU.

**Ablation experiments**

We have completed a comparative study using only NCCT imaging to predict HT in patients with acute ischaemic stroke. We compared the performance of the WSDL model with the ML models that used only NCCT feature information without clinical feature modelling, as shown in Figure 8 and Table 5. The code will be available at <https://github.com/TXVision/-Hemorrhagic_Transformation_Prediction.> Later, we further explored the features of these tricks.

**Table 5** Performances of WSDL model and ML models without clinical feature modelling

|  | **AUC** | **Accuracy** | **Sensitivity** | **Specificity** |
| --- | --- | --- | --- | --- |
| WSDL | 0.799(0.712,0.883) | 0.735(0.683,0.787) | 0.797(0.632,0.955) | 0.730(0.674,0.784) |
| baseline-DL | 0.748(0.638,0.856) | 0.776(0.729,0.826) | 0.638(0.441,0.833) | 0.789(0.741,0.839) |
| LR | 0.776(0.692,0.862) | 0.673(0.642,0.704) | 0.768(0.670,0.872) | 0.664(0.632,0.697) |
| XGBoost | 0.676(0.557,0.792) | 0.692(0.640,0.744) | 0.681(0.501,0.860) | 0.692(0.640,0.746) |
| SVM | 0.577(0.471,0.710) | 0.405(0.352,0.458) | 0.362(0.190,0.528) | 0.409(0.353,0.465) |
| RF | 0.661(0.547,0.771) | 0.637(0.585,0.690) | 0.696(0.510,0.876) | 0.632(0.578,0.687) |
| KNN | 0.601(0.537,0.663) | 0.478(0.445,0.510) | 0.754(0.638,0.864) | 0.453(0.419,0.486) |


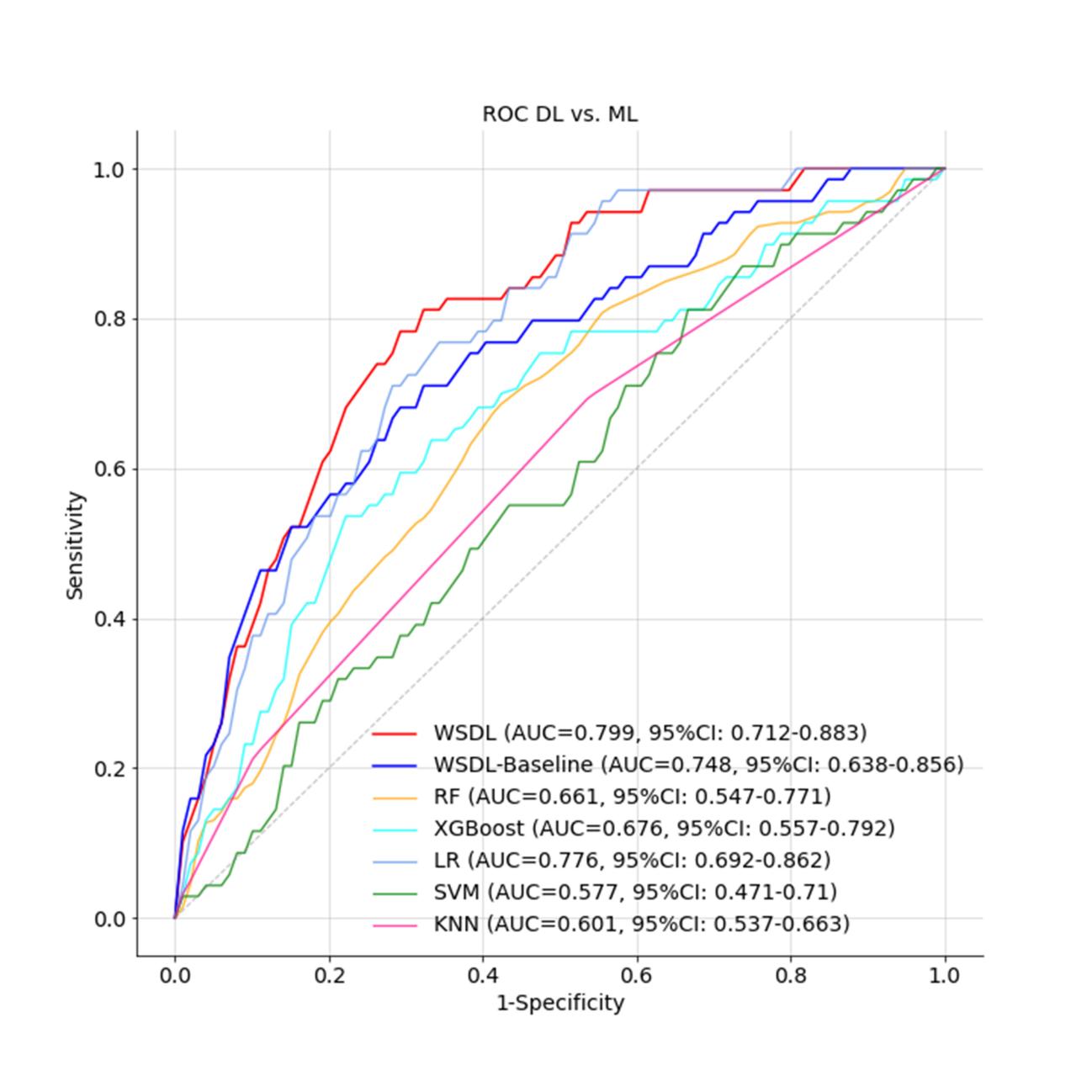


**Fig. 8** Illustration of the accuracy in terms of ROC curves for WSDL model and ML models that used only NCCT feature without clinical feature modelling

**REFERENCES**

1. Powers WJ, Rabinstein AA, Ackerson T, Adevoe OM, Bambakidis NC, Becker K, et al. 2018 guidelines for the early management of patients with acute ischaemic stroke: A guideline for healthcare professionals from the American Heart Association/American Stroke Association. Stroke. 2018;49(3):e46–99.
2. Deng J, Dong W, Socher R, Li LJ, Li K and Fei-Fei L. ImageNet: A large-scale hierarchical image database. 2009 IEEE Conference on Computer Vision and Pattern Recognition, Miami, FL, USA, 2009, pp 248–255. https://doi.org/10.1109/ CVPR.2009.5206848.
3. Hinton GE, Srivastava N, Krizhevsky A, Sutskever I, and Salakhutdinov RR. Improving neural networks by preventing co-adaptation of feature detectors. Comput Sci. 2012;3:212–23. https://doi.org/10.48550/arXiv.1207.0580.
